# World Organisation of Family Doctors Africa is moving!

**DOI:** 10.4102/phcfm.v11i1.2174

**Published:** 2019-09-19

**Authors:** Shabir Moosa

**Affiliations:** 1 Department of Family Medicine and Primary Care, Faculty of Health Sciences, University of the Witwatersrand, Johannesburg, South Africa; 2 World Organisation of Family Doctors (WONCA) Africa, Johannesburg, South Africa

Time has moved since my last briefing of readership in October 2018 on getting to know World Organisation of Family Doctors (WONCA) Africa.

There were a number of plans that the WONCA Africa Executive Committee (EXCO) set out in October 2018, and a number of achievements to date:

- *Increasing membership of WONCA Africa, especially academic departments of family medicine*: There has been considerable outreach to various countries that are not members of WONCA. We now have applications (for full and academic membership) being planned by Ethiopia, Botswana, the Democratic Republic of Congo, Cameroon and Somaliland. We have many more countries and expressions of interests which we need to follow up. We wish to see our membership footprint grow across all 46 countries in sub-Saharan Africa. We welcome any enquiries and suggest you visit the WONCA Africa website to see how your country can join and benefit.
- *Developing resources for academic departments including research collaborations*: There has been active collaboration and support of Primafamed, including engaging it as the ‘academic education/research’ wing of WONCA Africa. A few resources were also developed, for example, WONCA Africa PhD Forum and WONCA Talks. There is a wealth of possibilities that can be developed to support member organisations if there were to be a shared approach to academic development across Africa. I am actively advocating that learning materials for an online workplace-based Diploma in Family Medicine need to be more widely developed and shared across Africa so that we can develop family doctor-led primary health care (PHC) teams at scale to satisfy universal health coverage needs in Africa.
- *Improving women and young doctor participation in WONCA Africa and Member Organisations*: The Working Party on Women in Family Medicine has been active and had many activities in a preconference and during the Kampala Conference. Afriwon, as the young family doctor movement of Africa, has been active in online media with WhatsApp and Facebook groups, preconference and during the Kampala Conference. It is of vital importance that these groups are supported to continue and strengthen their activities between conferences.
- *Strengthening African involvement in WONCA working parties and special interest groups*: There has been a marked improvement with African members of WONCA working parties and special interest groups on rural health, mental health, ethics, emergency, education, research, etc., having workshops and presentations in the WONCA Africa Conference in Kampala. We must ensure that they do so identifiably in future, as part of the global network of WONCA working parties and special interest groups and with participation by global representatives.
- *Improving funding of WONCA Africa*: WONCA World has been very generous in supporting the African President with $7000 for African activities. Most of this is for travelling, but a part of this has been used for Zoom subscriptions, website domain, website hosting and other value-add for members of member organisations. We resolved many years ago to collect $1 per member of member organisations for WONCA Africa. This is finally being collected, with the assistance of WONCA World. There has been an attempt to raise donations for WONCA Africa, but this has proven difficult. We have received approval to develop a WONCA Africa fund (hosted by WONCA World) to provide vital seed-funding for future WONCA Africa conferences as this hurdle presents many difficulties to new and small member organisations wishing to host the WONCA Africa conference.
- *Adopting regional bylaws to ensure good governance of WONCA Africa*: The WONCA Africa Constitution adopted in October 2018 has been submitted to the WONCA Bylaws and recommendations presented to the WONCA Executive Committee Meeting in November 2019.
- *Improve communications to ordinary family doctors as well as member organisations*: There is a considerable amount of transparency now with reports and documents shared with EXCO members as well as member organisations. This is a culture we need to bake into our workings as the organisation of family doctors in Africa. We have been actively posting useful information on the WONCA Africa website (and www.ProfMoosa.com) as well as cross-posting on WONCA Africa groups or pages on Telegram, Facebook Page, Twitter, etc. There is a monthly email to a database of all family doctors interested in WONCA Africa. We are hoping that all member organisations will provide us their database of members so that we can provide them useful information from WONCA Africa and engage in continental debate on the future of family medicine.

Our priority was to ensure that the WONCA Africa Conference in Kampala, Uganda, 06–08 June 2019, was a success. It has been a success, indeed; 181 delegates from 32 countries came to Uganda to mingle with an array of speakers on the theme ‘People-centred PHC’. We were very fortunate in not only having the WONCA President Dr Donald Li and Immediate Past President Prof Amanda Howe present but also had high-level WHO (World Health Orginazation) representatives from Geneva and Brazzaville with us throughout the conference, with Dr Prosper Tumusiime, Acting Head of the WHO AFRO Health System Strengthening Cluster, opening and closing the conference. We ended the conference with the Kampala Commitment 2019: a commitment by family doctors to universal health coverage, primary healthcare and building the capacity of primary healthcare teams at scale in Africa. This is published in another article in this journal.

Dr Tumusiime was very impressed with family doctors of Africa, and we parted ways with firm plans on a way forward for a WHO–WONCA partnership in Africa. We also had Mr Nathaniel Otoo of Strategic Purchasing Africa Resource Centre (SPARC) present throughout and sharing his thoughts on provider payment reforms under universal health coverage in Africa. He also left with a SPARC–WONCA partnership firmly in mind. There will be more details on these plans in the next editorial.

Check the WONCA Africa website at www.woncaafrica.org, and @WONCAAfrica on Facebook, Twitter and Telegram to keep abreast of things at WONCA Africa.

